# Newly assembled mitochondrial genomes of *Hypericum* (Hypericaceae) provide insights into phylogenetic relationships and inter-organellar conflict

**DOI:** 10.3389/fpls.2026.1763082

**Published:** 2026-02-04

**Authors:** Jia-Xin Yu, Rui-Zhu Bai, Ya-Ping Chen, Xiao-Lei Ma, Ferhat Celep, Chun-Lei Xiang

**Affiliations:** 1 State Key Laboratory of Phytochemistry and Natural Medicines, Kunming Institute of Botany, Chinese Academy of Sciences, Kunming, China; 2 College of Life Sciences, University of Chinese Academy of Sciences, Beijing, China; 3 Yunnan Key Laboratory of Plant Diversity and Biogeography of East Asia, Kunming Institute of Botany, Chinese Academy of Sciences, Kunming, China; 4 Department of Biology, Faculty of Engineering and Natural Sciences, Kırıkkale University, Kırıkkale, Türkiye

**Keywords:** genomic evolution, *Hypericum*, inter-organellar conflict, Malpighiales, mitochondrial genome, phylogenomics

## Abstract

*Hypericum* is the largest genus in Hypericaceae and is widely recognized for its medicinal importance. To investigate the evolutionary dynamics of the genus, we assembled and annotated the first mitochondrial genomes of *H. ascyron* (411,519 bp) and *H. perforatum* (485,128 bp). Both genomes were recovered as circular structures and contained 30 conserved protein-coding genes (PCGs). Phylogenetic analyses based on 29 mitochondrial PCGs resolved Hypericaceae as sister to Podostemaceae, forming a distinct clade closely related to Calophyllaceae. Notably, we detected topological incongruence between mitochondrial and plastid phylogenies for *H. hirsutum*, *H. pulchrum*, and *H. perforatum*, indicating inter-organellar conflict within the genus. In addition, 316–324 RNA editing sites were predicted, many of which resulted in non-synonymous codon changes. These newly generated mitogenomes represent valuable genomic resources and provide new insights into the genetic diversity and evolutionary complexity of *Hypericum*.

## 1 Introduction

Mitochondria are essential energy-producing organelles in eukaryotic cells, generating adenosine triphosphate (ATP) through oxidative phosphorylation and contributing to a wide range of fundamental cellular processes (Møller et al., 2021; Borcherding and Brestoff, 2023; Monzel et al., 2023). The mitochondrial genome (mitogenome) retains genetic evidence of the organelle’s evolutionary origin and encodes several core functional proteins (Sloan et al., 2012; Roger et al., 2017; Wang et al., 2024). Plant mitogenomes exhibit remarkable variation in size, ranging from 66 kb in the parasitic plant *Viscum scurruloideum* Barlow (Santalaceae) to as large as 18.99 Mb in *Cathaya argyrophylla* Chun & Kuang (Pinaceae) (Skippington et al., 2015; Huang et al., 2025). Their structure organization is highly complex and dynamic; although often depicted as circular molecules, plant mitogenomes can also occur in linear or branched forms (Smith and Keeling, 2015; Kozik et al., 2019). Such structural diversity is largely driven by repeat-mediated recombination, which generates multiple alternative genomic conformations. For example, in *Morus* L., large repeats promote the coexistence of multiple mitogenome structures, resulting in pronounced structural heterogeneity (Liu et al., 2024a). Similarly, extensive alternative conformations and reticulated structures have been reported in *Gossypium* L. (Kong et al., 2025 ). and *Sorghum* Moench (Zhang et al., 2023). In addition, cytosine (C) to uracil (U) RNA editing is a widespread post-transcriptional modification in plant mitochondria (Small et al., 2020). Despite extensive variation in genome size and structure, protein-coding genes (PCGs) within plant mitogenomes remain generally conserved (Gualberto and Newton, 2017; Hu et al., 2025).

Obtaining pure mitochondria is technically challenging due to persistent plastid contamination and tissue-specific metabolites (Boussardon et al., 2020; Luo et al., 2020). Consequently, most plant mitogenomes are typically assembled from total genomic DNA, which often leads to the co-assembly of nuclear and plastid sequences (Li et al., 2025a; Ni et al., 2025; Shen et al., 2025). This process is further complicated by frequent intracellular gene transfer (IGT), resulting in the formation of nuclear mitochondrial transferred fragments (NUMTs) and mitochondrial plastid transferred fragments (MTPTs) (Wang et al., 2024). Third-generation sequencing platforms such as PacBio HiFi and Oxford Nanopore have therefore become indispensable for resolving complex repetitive regions and structural rearrangements in plant mitogenomes (Lian et al., 2024; Almeida and Marques, 2025).

*Hypericum* L. (Malpighiales), a diverse genus of herbs, shrubs, and small trees, comprises approximately 500 species, making it the largest genus in Hypericaceae (Nürk et al., 2013; APG IV, 2016). The genus is distributed globally, with major centers of diversity in Eurasia and the Andean region of South America (Nürk and Blattner, 2010; Meseguer et al., 2013; Nürk et al., 2013). In China, 75 species and 9 subspecies have been documented (Robson, 2012, 2016; Bai et al., 2023, 2025). Owing to their long history of medicinal use, several species of *Hypericum* are widely recognized for their pharmacological properties. Among them, *H. ascyron* L. and *H. perforatum* L. (Saint John’s wort) exhibits diverse pharmacological activities, including antitumor, antidepressant, anti-inflammatory, antiproliferative, antimicrobial, neuroprotective and antioxidant activities (Zhang et al., 2020; Vincent et al., 2021; Kapoor et al., 2023; Liu et al., 2024b). These species also serve as ingredients in cosmeceutical formulations and food products (Jarzębski et al., 2020; Jakubczyk et al., 2021; Silva et al., 2021).

Previous phylogenetic studies of *Hypericum* have relied primarily on nuclear ribosomal ITS and plastid markers (Meseguer et al., 2013; Nürk et al., 2013, 2015). Although Ruhfel et al. (2011) included a mitochondrial gene (*matR*) in phylogeny reconstruction of *Hypericum* within the clusioid clade, using combined plastid and mitochondrial sequences, single-gene mitochondrial data provide limited insight into mitogenome evolution. Phylogenetic incongruence among nuclear, plastid and mitochondrial genomes is common in land plants, and may arise from processes such as incomplete lineage sorting (ILS) and hybridization, or analytical artifacts (Cox, 2018; Sousa et al., 2020). Recent mitogenome studies in large genera such as *Dendrobium* Sw. (Orchidaceae) and *Quercus* L. (Fagaceae) have revealed substantial cytonuclear discordance (Wang et al., 2023; Song et al., 2025). Given their low nucleotide substitution rates (Chen et al., 2017) and uniparental inheritance (Hu et al., 2025), plant mitogenomes provide an independent line of evidence for testing phylogenetic conflict. Furthermore, mitochondrial phylogenomics has yielded new insights into deep angiosperm relationships (Xue et al., 2022). As a species-rich and widely distributed lineage, *Hypericum* may likewise exhibit complex evolutionary patterns, underscoring the need for comprehensive mitogenome analyses.

In this study, we assembled and characterized the complete mitochondrial genomes of *H. ascyron* and *H. perforatum* using PacBio high-fidelity (HiFi) long-read sequencing. By integrating these newly generated mitogenomes with four additional *Hypericum* mitogenomes obtained from the Darwin Tree of Life (DToL) Project (The Darwin Tree of Life Project Consortium, 2022), we performed the first comparative mitogenomic analysis of this genus, with a particular focus on RNA editing, genome collinearity, and phylogenetic relationships. In addition, we constructed a mitochondrial phylogenetic framework for Malpighiales based on conserved mitochondrial PCGs. Collectively, our results provide foundational mitochondrial genomic resources for *Hypericum*, improve understanding of its evolutionary history within Malpighiales, and offer new perspectives for future breeding and evolutionary studies.

## 2 Materials and methods

### 2.1 Plant materials and sequencing

*Hypericum ascyron* and *H. perforatum* were cultivated in the greenhouse of the Kunming Institute of Botany, Chinese Academy of Sciences. The plants were originally transplanted from Anlong County (24°58′ E, 105°35′ N) and Fenggang County (27°39′ E, 107°43′ N), Guizhou Province, China, respectively. Fresh young leaves were collected, immediately frozen in liquid nitrogen and stored at –80°C.

Total genomic DNA was extracted using the modified cetyltrimethylammonium bromide (CTAB) protocol (Doyle and Doyle, 1987). The standard HiFi sequencing libraries were prepared with the SMRTbell Express Template Prep Kit 2.0 (Pacific Biosciences), and sequencing was performed on the PacBio Revio platform by Wuhan Frasergen Bioinformatics Co., Ltd. (Wuhan, China). For each species, approximately 10 Gb of PacBio HiFi data were generated, with total read numbers ranging from 516,416 to 632,999 and mean read lengths of 17,234 to 19,075 bp (**Supplementary Table S1**).

### 2.2 Genome assembly and annotation

Initial mitochondrial genome assemblies were generated using four PacBio HiFi read using four assemblers: HiMT v.1.1.0 (Tang et al., 2025), Oatk v.1.0 (Zhou et al., 2025), PMAT2 v.2.1.5 (Han et al., 2025) and TIPPo v.2.4 (Xian et al., 2025). These assemblers employ complementary strategies, including read filtering (HiMT, TIPPo) and graph-based resolution (Oatk, PMAT2). Parameters were set as follows: HiMT (default), Oatk (-k 1001 -c 50 -m embryophyta_mito.fam), TIPPo (-p hifi --trf), and PMAT2 (“autoMito” mode with estimates of 700 Mb for *H. perforatum* and 500 Mb for *H. ascyron*).

Plastid genomes of *H. ascyron* and *H. perforatum* were also assembled from HiFi reads using Oatk v.1.0, with the specialized “embryophyta_pltd.fam” gene profile. Assembly graphs were visualized and examined using Bandage v.0.8.1 (Wick et al., 2015). Based on structural comparative assessment of assembly and contiguity metrics (**Supplementary Table S2**), the HiMT-derived assemblies were selected as the final mitogenomes. These assemblies were subsequently validated for continuity and completeness. Assembly completeness was evaluated with the HiMT “assess” module by detecting conserved mitochondrial genes.

Raw HiFi reads were mapped to the draft mitogenomes using minimap2 v.2.28-r1209 (Li, 2021) with the “-ax map-hifi” parameters to evaluate continuity. Coverage depth was calculated with samtools coverage v.1.15.1 (Li et al., 2009) and bamtocov v.2.2.0 (Birolo and Telatin, 2022) and visualized using custom python script.

Mitogenomes and plastomes of *H. androsaemum* L., *H. hircinum* L., *H. hirsutum* L. and *H. pulchrum* L. were retrieved as unannotated assemblies from the Darwin Tree of Life (DToL) Project (The Darwin Tree of Life Project Consortium, 2022); the corresponding accession numbers are provided in **Supplementary Tables S3** and **S4**. Mitochondrial genomes were annotated using the online tool PMGA (Li et al., 2025b), with the “29 Mitogenomes” database, whereas plastomes were annotated using PGA v.2.0 (Zhang et al., 2025). All annotation were manually curated in Geneious Prime v.2025.0.2 (Kearse et al., 2012) using closely related mitogenomes (e.g., *Terniopsis yongtaiensis* X.X.Su, Miao Zhang & B.Hua Chen OR818323, *Calophyllum soulattri* Burm.f. NC_079842) and plastomes (e.g., *Cratoxylum arborescens* (Vahl) Blume NC_062807, *Vismia macrophylla* Kunth PX719231). Circular genome maps were generated with OGDRAW v.1.3.1 (Greiner et al., 2019), and trans-splicing gene structures were visualized using PMGmap (Zhang et al., 2024c).

### 2.3 Repeat sequences and codon usage bias analysis

Three types of repeat sequences were identified: simple sequence repeats (SSRs), dispersed repeats, and tandem repeats. SSRs were detected with MISA v.2.1 (Beier et al., 2017) using ‘1-10 2-5 3-4 4-3 5-3 6-3’ thresholds. Dispersed repeats were identified with REPuter (Kurtz, 2001) using parameters ‘-c -f -p -r -l 30 -h 3 -best 5000’. Tandem repeats were detected using Tandem Repeats Finder v.4.09 (Benson, 1999) with default settings. The distribution and frequency of repeat sequences were visualized in R using ggplot2 (Wickham, 2016) and circlize (Gu et al., 2014) package.

Protein-coding genes (PCGs) were extracted using PhyloSuite v.1.2.3 (Xiang et al., 2023) and relative synonymous codon usage (RSCU) values were calculated using CodonW v.1.4.2 (https://codonw.sourceforge.net/).

### 2.4 Identification of intracellular transferred sequences

Homologous regions between mitochondrial and plastid genomes were identified using BLASTN v.2.16.0 (Camacho et al., 2009) with an E-value cutoff of 1e-5. Only alignments with a minimum length of 30 bp and a sequence identity of at least 70% were retained for subsequent analyses. The identified mitochondrial plastid transferred fragments (MTPTs) were visualized using the R package circlize.

### 2.5 Comparative analysis of six *Hypericum* mitochondrial genomes

Six *Hypericum* mitogenomes (two newly assembled and four from DToL) were compared. Gene copy number variation was visualized using heatmaps. RNA editing sites were predicted from PCGs using Deepred-Mt (Edera et al., 2021) with default settings, retaining sites with prediction probability values ≥ 0.9. Results were plotted using ggplot2 package.

Mitogenomes of *Calophyllum soulattri* (NC_079842) and *Terniopsis yongtaiensis* (OR818323), and plastomes of *Cratoxylum arborescens* (NC_062807) and *Vismia macrophylla* (PX719231) were retrieved from National Center for Biotechnology Information (NCBI) (**Supplementary Tables S3**-**S4**), and used as outgroups. A total of 30 mitochondrial PCGs and 71 plastid PCGs shared among taxa were aligned with MAFFT v.7.505 (Katoh and Standley, 2013) using the ‘--auto’ strategy and codon alignment mode. Gaps were removed with trimAl v.1.2rev57 (Capella-Gutiérrez et al., 2009) using “-automated1”, and concatenation was performed in Phylosuite (Xiang et al., 2023).

Phylogenetic trees were reconstructed using RAxML-NG v.1.2.2 (Kozlov et al., 2019) under the best-fit substitution models determined by ModelFinder v.2.2.0 (Kalyaanamoorthy et al., 2017) based on the Akaike Information Criterion (AIC): “GTR+F+G4” for mitogenomes and “GTR+F+I+G4” for plastomes. Maximum likelihood (ML) analyses used the “--all --bs-trees 5000” parameters, phylogenetic trees were visualized and edited using Figtree v.1.4.5 (http://tree.bio.ed.ac.uk/software/figtree/).

Pairwise synteny analysis among *Hypericum* mitogenomes was conducted using the “GetTwoGenomeSyn.pl” script of NGenomeSyn v.1.41 (He et al., 2023) with integrated minimap2. Homologous regions longer than 5,000 bp were retained as conserved colinear blocks for visualization and analysis.

### 2.6 Phylogenetic analysis for Malpighiales

To determine the phylogenetic position of *Hypericum* within Malpighiales, 19 published mitogenomes from seven families (Calophyllaceae, Clusiaceae, Euphorbiaceae, Passifloraceae, Podostemaceae, Rhizophoraceae and Salicaceae) were retrieved from NCBI. Five species from Celastrales and Sapindales were selected as outgroups (**Supplementary Table S3**). Twenty-nine shared mitochondrial PCGs were extracted in PhyloSuite v.1.2.3 (Xiang et al., 2023) and analyzed using the same ML method described above under the best-fit model “GTR+F+I+G4”.

Bayesian inference (BI) analyses were conducted in MrBayes v.3.2.7a (Ronquist et al., 2012) using two independent runs of 2000000 generations, sampling every 1,000 generations. The first 25% of samples were discarded as burn-in, and convergence was confirmed by an average standard deviation of split frequencies < 0.01.

## 3 Results

### 3.1 Genome assembly and general features of *H. ascyron* and *H. perforatum*

Using approximately 9.8–10.9 Gb of Pacbio HiFi long reads (**Supplementary Table S1**), we assembled the mitochondrial genomes of *H. ascyron* and *H. perforatum* with four assemblers. Although minor differences in genome topology and contiguity were observed (**Supplementary Figure S1**; **Supplementary Table S2**), these discrepancies were largely attributed to differences in how long repeat sequences were resolved by respective algorithms.

**Figure 1:**
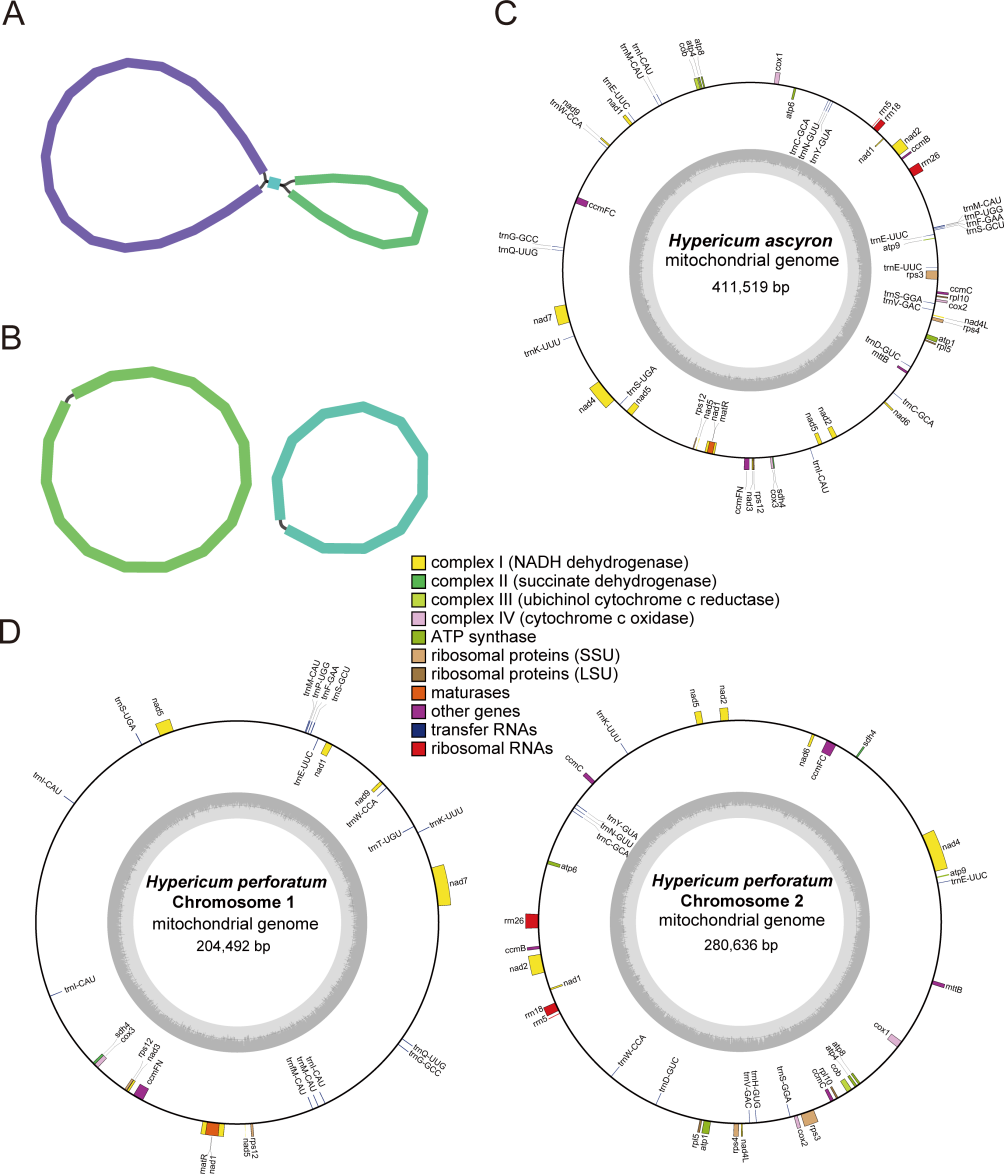
Mitogenome assembly graphs and annotation maps of *H. ascyron* and *H. perforatum*. **(A)** Assembly graph of the *H. ascyron* mitogenome. **(B)** Assembly graph of the *H. perforatum* mitogenome. **(C)** Annotated circular map of the *H. ascyron* mitogenome. **(D)** Annotated circular map of the *H. perforatum* mitogenome. Genes belonging to different functional categories are indicated by distinct colors. The inner circle represents the GC content (%).

As shown in **Supplementary Figure S1**, the assemblies of *H. perforatum* generated by HiMT, Oatk, and TIPPo were highly consistent, each producing two contigs that formed a typical circular mitogenome. In contrast, all assemblers produced a single circular molecule for *H. ascyron*, with one or two repeat regions (3,302–5,233 bp) inferred from doubled coverage depth relative to unique contigs. Owing to its superior contiguity and well-resolved assembly graph topology, the HiMT assembly was selected as the final mitochondrial genome for both species (**Figures 1A, B**).

Mapping back the HiFi reads confirmed consistent coverage depth, i.e. 456.47× for *H. ascyron*, 470.17× for *H. perforatum* Chr1, and 950.57× for *H. perforatum* Chr2 (**Supplementary Figure S2**). The mitogenome lengths were 411,519 bp for *H. ascyron* and 485,128 bp for *H. perforatum*. The latter comprises two circular chromosomes of 204,492 bp (Chr1) and 280,636 bp (Chr2) (**Supplementary Table S1**). The plastomes of *H. ascyron* and *H. perforatum* were 163,564 bp and 139,734 bp in length, respectively. Both exhibited a typical quadripartite structure consisting of a large single-copy (LSC) region and a small single-copy (SSC) region, separated by a pair of inverted repeat (IR) regions (**Supplementary Figure S3**).

The length of *Hypericum* mitogenomes ranged from 411,519 bp to 494,351 bp with GC contents of 43.92–44.28% (**Supplementary Table S5**). Gene content showed moderate variation among species, with 30 unique PCGs and 17–21 tRNAs identified (**Table 1**). Several mitochondrial genes contained multiple introns. Specifically, *nad1*, *nad2*, and *nad5* were trans-spliced (**Supplementary Figure S4**), whereas *ccmFC*, *nad4*, *nad7* and *rps3* underwent cis-splicing. Plastid genome lengths ranged from 139,437 to 165,518 bp, with GC content of 37.3–37.7%, and encoded 75–79 PCGs, 34–35 tRNAs, and 8 rRNAs (**Supplementary Table S6**).

**Table 1:** Gene composition of the six *Hypericum* mitogenomes.

Gene category	Functional category	Gene name
Core genes	ATP synthase	*atp1*, *atp4*, *atp6*, *atp8*, *atp9*
	Cytochrome c biogenesis	*ccmB*, *ccmC*, ccmFC*, *ccmFN*
	Ubiquinol cytochrome c reductase	*cob*
	Cytochrome c oxidase	*cox1*, *cox2*, *cox3*
	Maturases	*matR*
	Transport membrane protein	*mttB*
	NADH dehydrogenase	nad1****, nad2****, *nad3*, nad4***, *nad4L*, nad5****, *nad6*, nad7****, *nad9*
Variable genes	Large subunit of ribosome	*rp110*, *rp15*
	Small subunit of ribosome	*rps12*, rps3*, *rps4*
	Succinate dehydrogenase	*sdh4*
	Ribosomal RNAs	*rrn18*, *rrn26*, *rrn5*
	Transfer RNAs	*trnC-GCA*, *trnD-GUC*, *trnE-UUC*, *trnF-GAA*, *trnG-GCC*, *trnH-GUG*, *trnI-CAU*, *trnK-UUU*, *trnM-CAU*, *trnN-GUU*, *trnP-UGG*, *trnQ-UUG*, *trnR-UCU*, *trnS-GCU*, *trnS-GGA*, *trnS-UGA*, *trnT-UGU*, *trnV-GAC*, *trnW-CCA*, *trnY-GUA*, *trnfM-CAU*

*intron number

### 3.2 Repeat sequences in the mitogenomes of *H. ascyron* and *H. perforatum*

**Figure 2:**
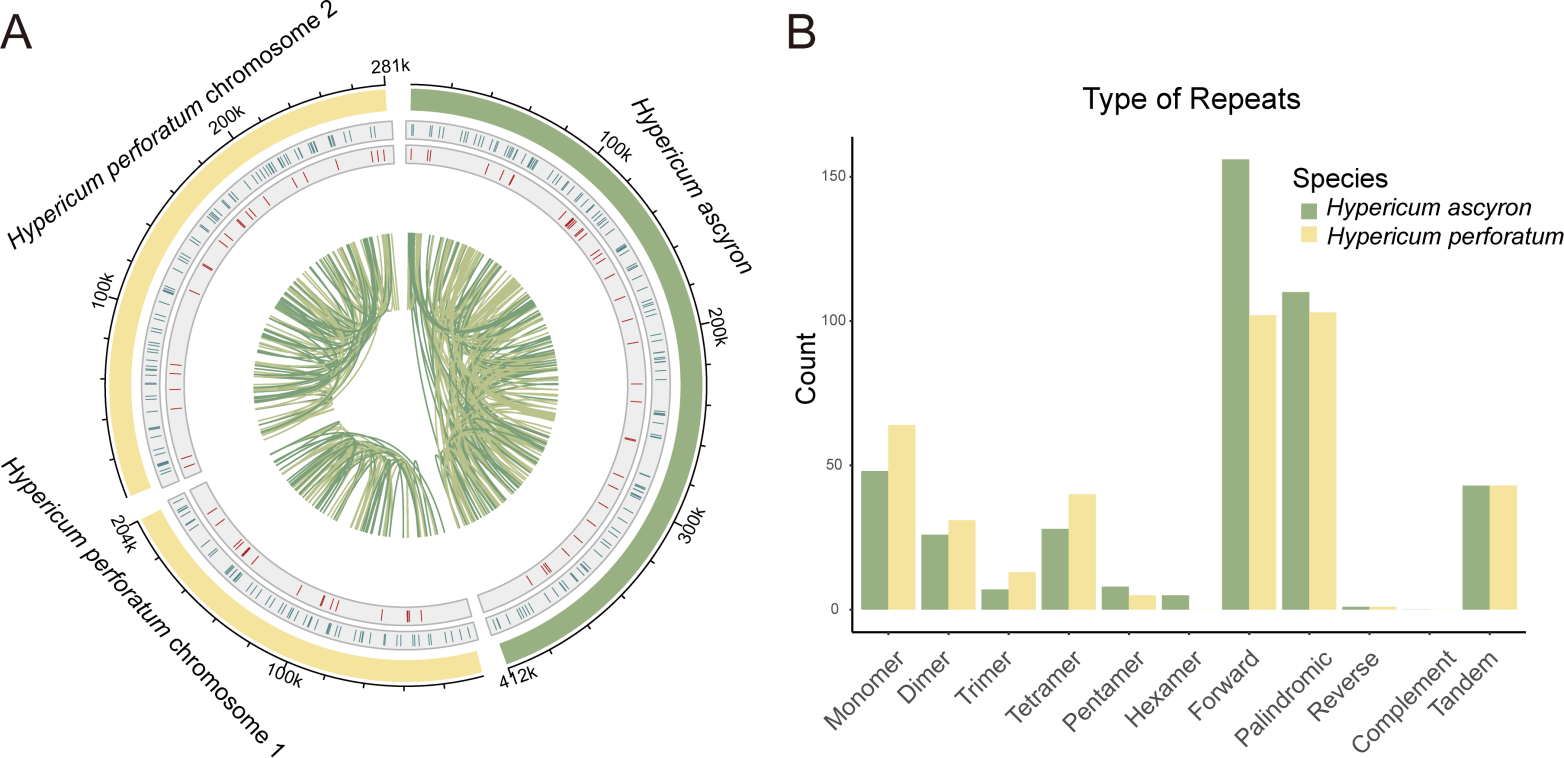
Repeated sequences identified in the *H. ascyron* and *H. perforatum* mitogenomes. **(A)** Distribution of repetitive elements. The innermost circle shows dispersed repeats, the middle circle displays tandem repeats (red bars), and the outermost circle depicts SSRs (blue bars). The scale on the outermost circle is marked at 20-kb intervals. **(B)** Frequency of SSRs, tandem repeats, and dispersed repeats. The x-axis represents repeat types, and the y-axis indicates the number of repeats detected.

Three classes of repetitive sequences were identified: simple sequence repeats (SSRs), tandem repeats, and dispersed repeats. Their distribution varied markedly across the genomes (**Figure 2A**). SSRs were broadly and evenly distributed, whereas tandem repeats occurred less frequently. Dispersed repeats were the most abundant, dominated by short-distance events, with only a few long-distance pairs. Summary statistics for all repeat categories are presented in **Supplementary Table S7** and **Figure 2B**.

We detected 122 SSRs in *H. ascyron* and 153 in *H. perforatum*, totaling 1,630 bp (0.40%) and 2,117 bp (0.44%), respectively. These SSRs ranged from mono- to hexameric motifs, with mono-, di-, and tetrameric most prevalent. Hexameric SSRs were absent from *H. perforatum* but occurred five times in *H. ascyron*. Both species contained 43 tandem repeats; however, *H. perforatum* had a greater total tandem repeat length (2,616 bp) than *H. ascyron* (2,213 bp).

Dispersed repeats were classified as forward, palindromic, reverse, and complementary (Kurtz, 2001). Forward and palindromic repeats predominated in both species. *Hypericum ascyron* exhibited more dispersed repeats (267) than *H. perforatum* (206), corresponding 5.70% and 2.95% of the mitogenome, respectively. Both species contained one reverse repeat and no complementary repeats.

### 3.3 Codon usage bias analysis

**Figure 3:**
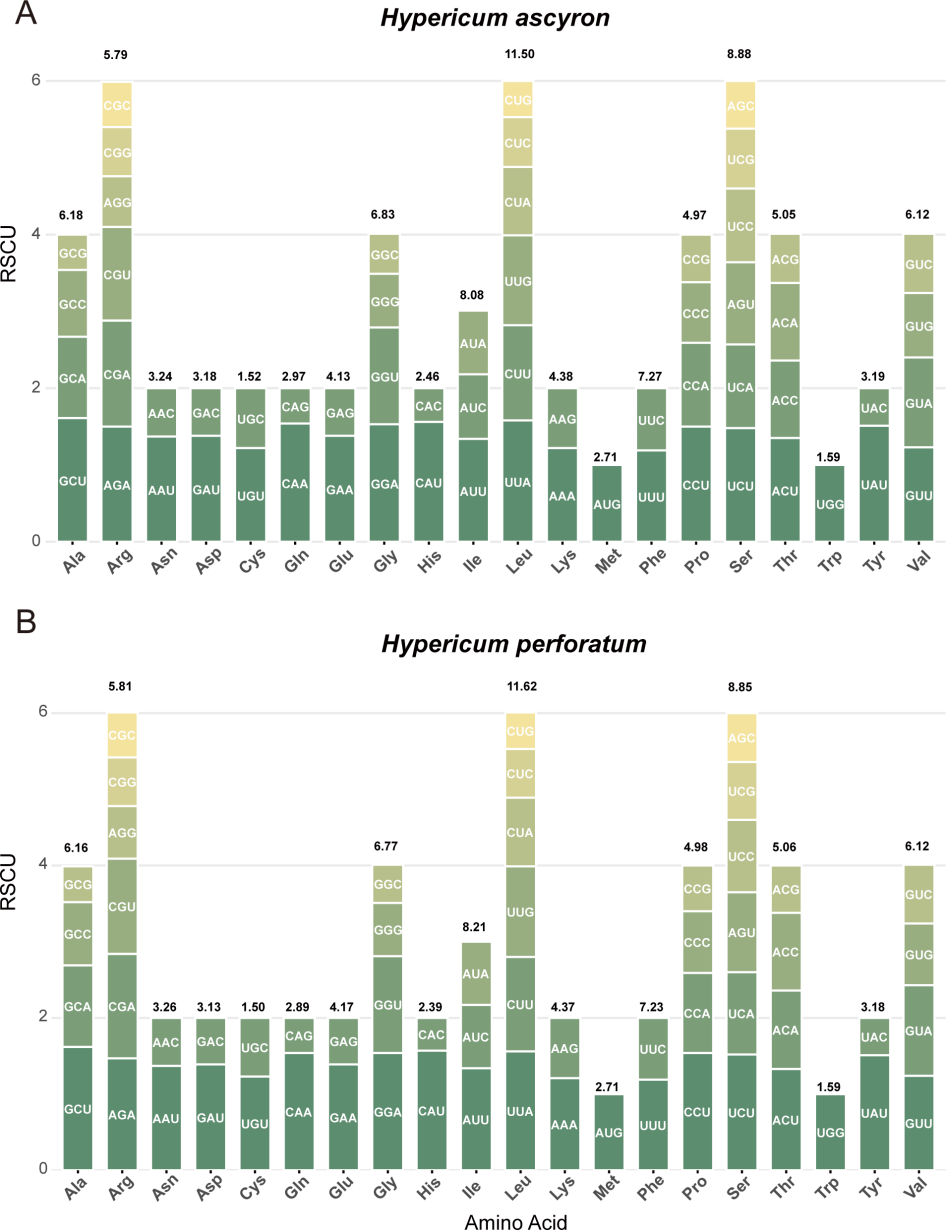
Relative synonymous codon usage (RSCU) patterns in mitochondrial protein-coding genes of **(A)***H. ascyron* and **(B)***H. perforatum*. The x-axis represents amino acids, the y-axis indicates RSCU value. Codons encoding the same amino acid are displayed as histogram in different colors. Amino acid usage values are indicated above the corresponding bars.

Codon usage was analyzed across 31 and 33 mitochondrial PCGs from *H. ascyron* and *H. perforatum*, respectively (**Figure 3**). The genes used all 64 possible codons, representing 20 amino acids. A total of 32 codons showed relative synonymous codon usage (RSCU) values ≥ 1 in both species, indicating preferential codon usage (**Supplementary Tables S8**-**S9**). Three stop codons (UAA, UGA, and UAG) were present. Among all codons, GCU (Ala) was the most frequently used, whereas CAC (His) was the least. Leucine (Leu) was the most abundant amino acid encoded.

### 3.4 Identification of mitochondrial plastid transferred fragments

**Figure 4:**
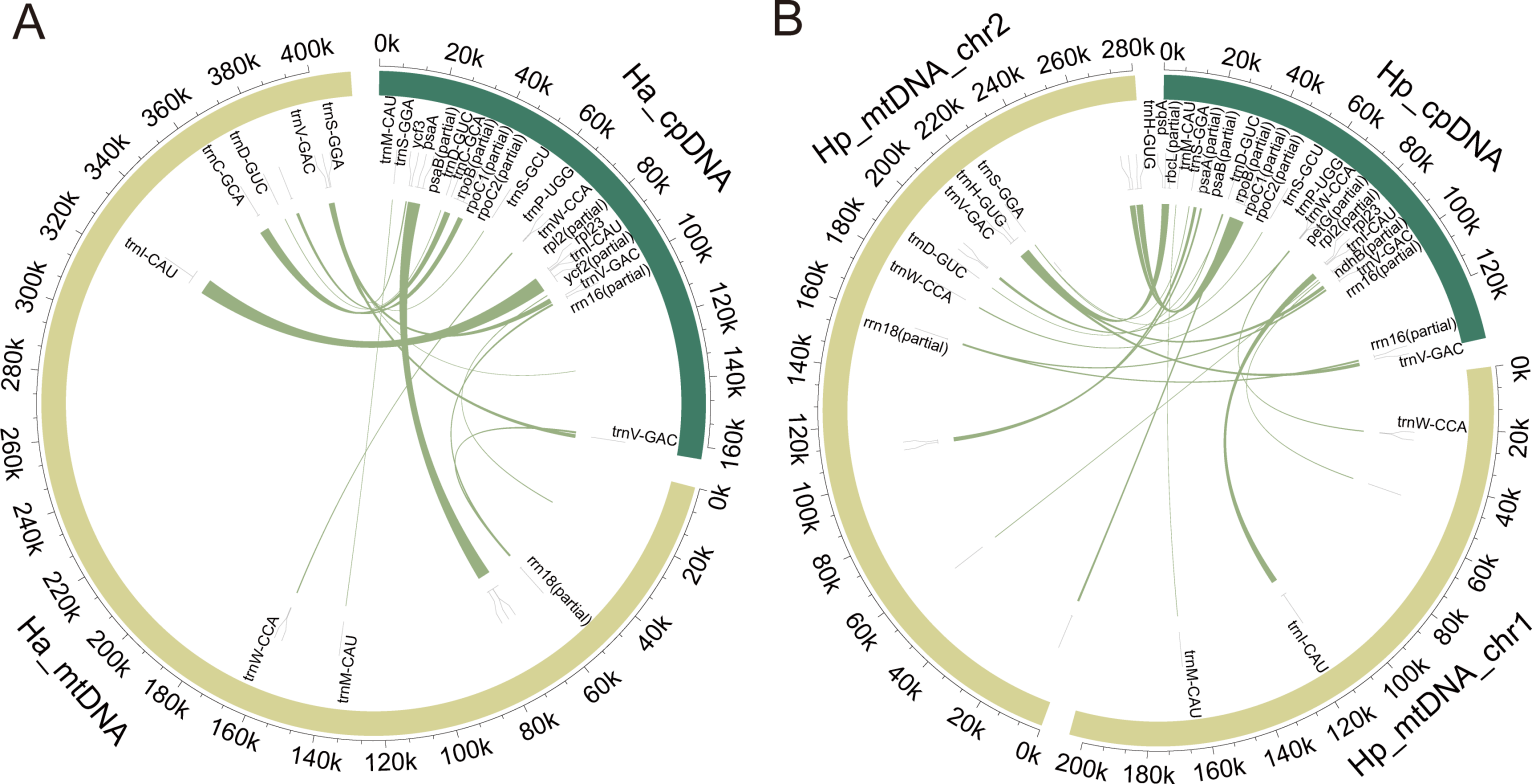
Homologous sequences identified between the plastomes and mitogenomes of **(A)***H. ascyron* and **(B)***H. perforatum*. Dark green and light green circles represent the plastome and mitogenome, respectively. The inner arcs show the homologous DNA fragments, with corresponding annotations shown along each arc.

We detected 22 plastid-derived fragments (Ha_MTPT 1–22) in *H. ascyron* and 25 (Hp_MTPT 1–25) in *H. perforatum* (**Figure 4**). MTPTs ranged from 39 bp to 6,337 bp and accounted for 5.9% of the *H. ascyron* and 4.6% in *H. perforatum* (**Supplementary Tables S10**-**S11**). Twelve MTPTs overlapped coding regions, collectively containing seven intact plastid tRNA genes and one partial *rrn18* sequence. The longest MTPT in *H. ascyron* (Ha_MTPT-1) included a full-length of *trnI-CAU* gene, which was also present in the second-longest MTPT of *H. perforatum* (Hp_MTPT-1; positions 113,185–115,949 bp on chr 1).

### 3.5 Comparative mitogenomic analysis among *Hypericum* species

**Figure 5:**
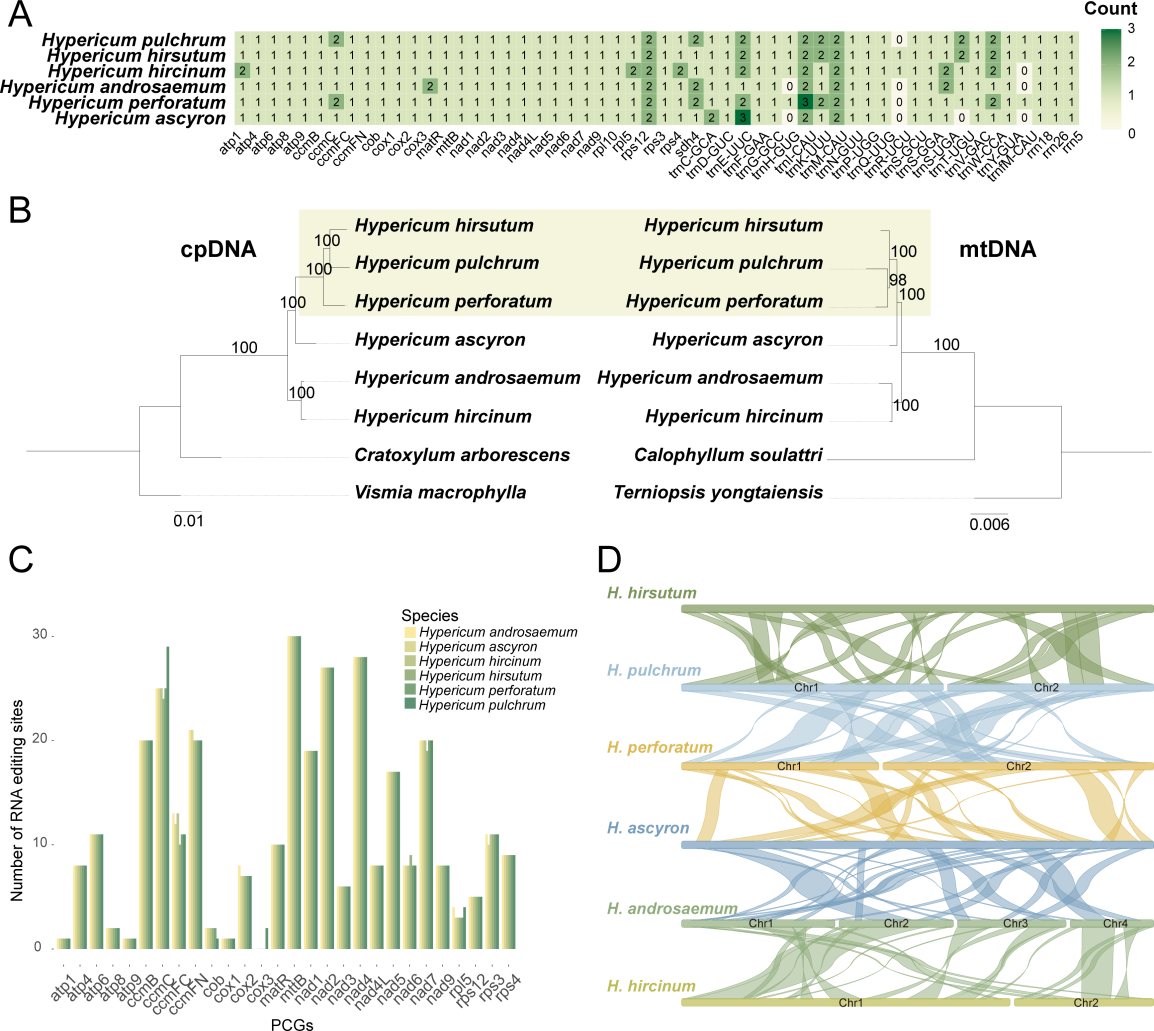
Integrated comparative analyses of six *Hypericum* mitogenomes, including four retrieved from the Darwin Tree of Life project. **(A)** Distribution patterns of mitochondrial PCGs, rRNA, and tRNA genes across *Hypericum* species. Color intensity corresponds to gene copy number. **(B)** Maximum-likelihood (ML) phylogenies of six *Hypericum* species inferred from 71 shared plastid genes (left) and 30 shared mitochondrial PCGs (right). Numbers at nodes represent bootstrap support values. Topological conflicts between the mitochondrial and plastid trees are highlighted with green squares. **(C)** Distribution of C-to-U RNA editing sites in mitochondrial PCGs among the six *Hypericum* species. The x-axis denotes gene names, and the y-axis indicates the number of RNA editing sites. **(D)** Colinear blocks among the six *Hypericum* mitogenomes. Rectangles represent the mitogenomes of each species.

Comparisons among six *Hypericum* mitogenomes revealed variation in gene copy number (**Figure 5A**). All species shared a core set of 50 genes (30 PCGs, 17 tRNAs, and 3 rRNAs). Most PCGs were single-copy, but several genes showed species-specific duplications. *trnM-CAU* and *rps12* were duplicated in all species, *atp1*, *rpl5*, and *rps4* were duplicated only in *H. hircinum*, and *cox3* and *trnC-GCA* were duplicated in *H. androsaemum* and *H. ascyron*, respectively. *trnE-UUC* and *trnI-CAU* had three copies in *H. ascyron* and *H. perforatum*, but one or two copies in other species. Several tRNAs (*trnH-GUG*, *trnR-UCU*, *trnT-UGU*, and *trnfM-CAU*) were absent from some mitogenomes (**Figure 5A**).

Phylogenetic relationships inferred from shared mitochondrial and plastid PCGs consistently recovered *Hypericum* as monophyletic (**Figure 5B**; ML-BS = 100%). Both datasets supported three clades: *H. androsaemum* + *H. hircinum*, *H. ascyron*, and *H. perforatum + H. hirsutum + H. pulchrum*. While both phylogenies placed *H. androsaemum* + *H. hircinum* as sister to all remaining species, they differed in resolving relationships among *H. hirsutum*, *H. pulchrum*, and *H. perforatum* (**Figure 5B**). The plastome-based phylogeny resolved *H. hirsutum + H. pulchrum* as a clade, whereas mitogenome data grouped *H. pulchrum* with *H. perforatum* (**Figure 5B**).

C-to-U RNA editing predictions across 28 shared PCGs identified 316–324 C-to-U editing sites per species (probability ≥ 0.9) (**Figure 5C**). *mttB* contained the most editing sites (30), followed by *ccmC*, *ccmFN*, *nad2* and *nad4* (each > 20). Editing profiles were largely conserved except for *ccmC* (five additional sites in *H. pulchrum*) and *cox3*, which was edited only in *H. pulchrum*. Of all predicted edits, 28–30 were synonymous, while the remainder were non-synonymous, corresponding to 11 types of amino acid changes (**Supplementary Tables S12**-**S17**). Most edits occurred at the second position (186–192), followed by the first (107–112) and the third (20–24).

Synteny analysis of homologous blocks > 5,000 bp revealed extensive rearrangements among species (**Figure 5D**). The largest conserved collinear block occurred between *H. androsaemum* Chr2 and *H. hircinum* Chr1, and the largest inverted block was found between *H. pulchrum* Chr1 and *H. perforatum* Chr1 (**Supplementary Table S18**).

### 3.6 Phylogenetic analysis of *Hypericum* within Malpighiales

**Figure 6:**
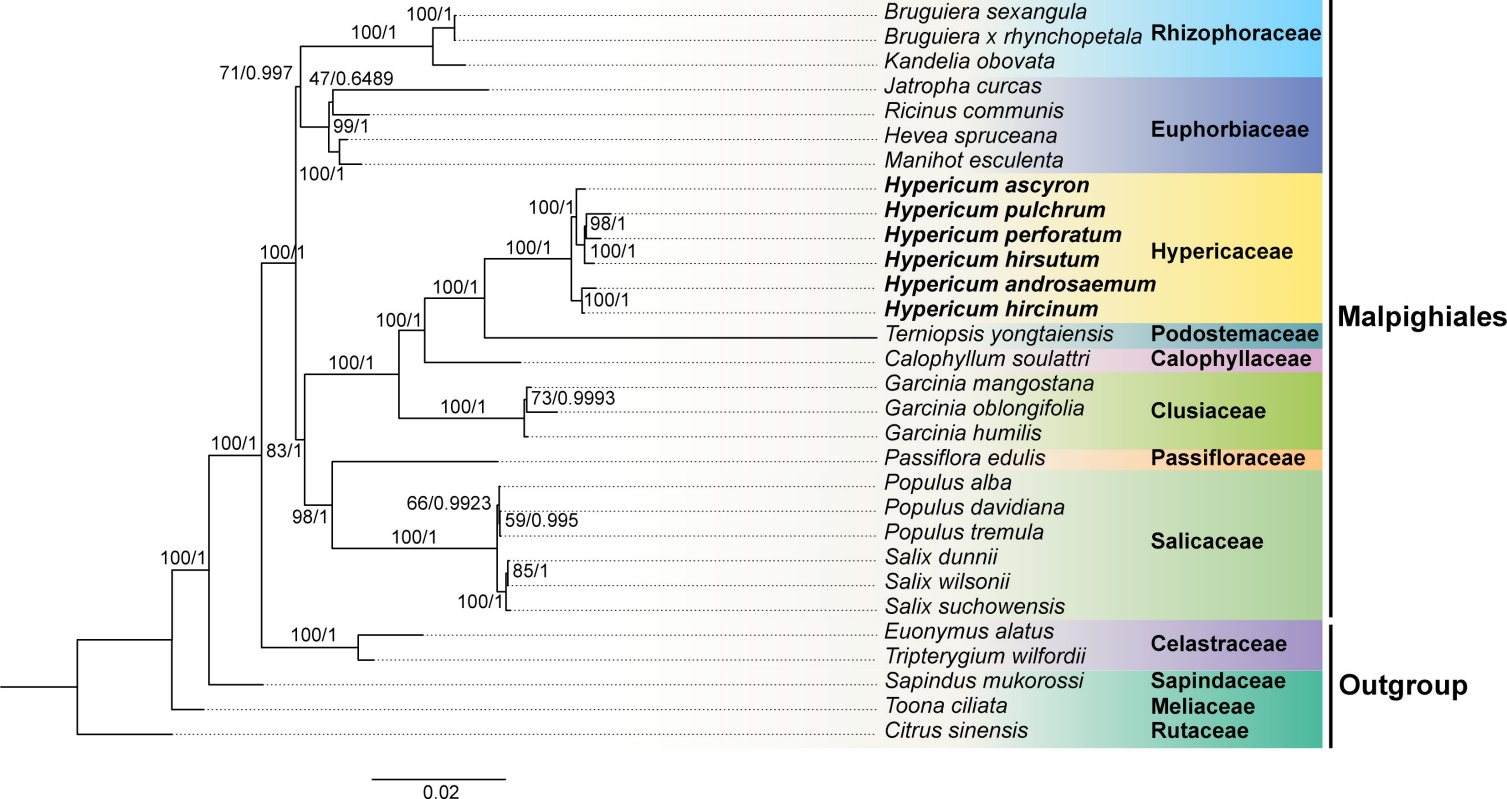
Phylogenetic tree of *Hypericum* and 19 additional Malpighiales species reconstructed from 29 conserved mitochondrial PCGs. Numbers at nodes indicate the bootstrap support values (ML-BS) and posterior probabilities (BI-PP). Different colors denote distinct taxonomic groups.

To clarify the evolutionary placement of *Hypericum* within Malpighiales, we reconstructed phylogenies using 29 conserved mitogenome PCGs from six *Hypericum* species and 24 representatives of Malpighiales, Celastrales and Sapindales. Both Maximum likelihood (ML) and Bayesian inference (BI) analyses strongly supported the monophyly of Malpighiales (**Figure 6**), which comprised three major clades: Rhizophoraceae + Euphorbiaceae, Passifloraceae + Salicaceae, and Hypericaceae + Calophyllaceae + Podostemaceae + Clusiaceae (**Figure 6**). Within the latter clade, Clusiaceae formed a sister group to the lineage including Calophyllaceae, Hypericaceae, and Podostemaceae, with Hypericaceae and Podostemaceae recovered as sister families.

## 4 Discussion

### 4.1 Structural and size variation of *Hypericum* mitogenomes

Plant mitochondrial genomes (mitogenomes) are characterized by exceptional structural complexity, which is largely driven by extensive repeat sequences and frequent recombination events (Yurina and Odintsova, 2016). In this study, we obtained complete and well-supported mitochondrial genome assemblies for *Hypericum ascyron* and *H. perforatum* by integrating multiple assembly strategies. Depth-of-coverage assessment and the HiMT “assess” module indicated that all genomic regions in the final assemblies were robustly supported (**Supplementary Figure S2**). Both mitogenomes contained the full set of 24 core mitochondrial protein-coding genes (PCGs) (Mower et al., 2012; Ni et al., 2025; Tang et al., 2025; **Table 1**), consistent with findings in other Malpighiales species such as *Calophyllum soulattri* (Cadorna et al., 2024) and *Terniopsis yongtaiensis* (Zhang et al., 2024b), and suggesting a relatively stable mitochondrial gene repertoire in this order compared with the extensive gene loss observed in *Viscum* L. (Petersen et al., 2015; Skippington et al., 2015).

As of November 2025, mitochondrial genomes have been reported for only about a dozen species within Malpighiales, revealing substantial size variation ranging from 371,235 bp in *Garcinia mangostana* L. (Wee et al., 2022 ). to 1,402,206 bp in *Hevea camargoana* Pires (Niu et al., 2024). Pronounced mitogenome size variation has also been documented in other angiosperm genera, such as *Dendrobium* Sw. (Soe et al., 2025). Despite this broad size range, the number of PCGs remains highly conserved, ranging from 29 in *G. mangostana* to 36 in *Banisteriopsis caapi* (Spruce ex Griseb.) C.V.Morton (Chavarro-Mesa et al., 2024) and *Viola diffusa* Ging (Zhang et al., 2024a). In this study, the mitogenomes of *H. ascyron* (411,519 bp) and *H. perforatum* (485,128 bp) fall within the lower-to-medium range of Malpighiales and are comparable to the closely related *Terniopsis yongtaiensis* (426,928 bp; Zhang et al., 2024b), but significantly larger than those of *Calophyllum soulattri* (378,262 bp; Cadorna et al., 2024) and *G. mangostana* (371,235 bp).

Although both *Hypericum* mitogenomes were assembled as circular molecules, previously deposited *Hypericum* assemblies from the DToL project remain as fragmented scaffolds (1–4 contigs; **Supplementary Table S5**), suggesting that multichromosomal structures may also occur within the genus (Wu et al., 2022). GC contents were highly conserved between the two species (43.9–44.2%) and closely matched other Malpighiales, such as *Hevea* Aubl. (44.13–44.24%) and *C. soulattri* (43.97%), highlighting stability in nucleotide composition despite considerable structural dynamics (Gualberto and Newton, 2017).

The ~74 kb size difference between *H. ascyron* and *H. perforatum* did not correspond to differences in unique gene content, supporting the widely accepted hypothesis that mitogenome expansion in angiosperms is primarily driven by increases in non-coding sequences, including repeats and foreign sequences acquired through intracellular gene transfer (MTPTs/NUMTs) or horizontal gene transfer (Chen et al., 2017; Wu et al., 2022). For example, size variation in Cucurbitaceae mitogenomes (390 kb to 2.9 Mb) is largely attributable to the accumulation of short dispersed repeats and plastid sequences (Alverson et al., 2010), whereas the extraordinary expansion in *Amborella trichopoda* Baill. mitogenome is primarily driven by extensive horizontal gene transfer (Rice et al., 2013). In *Hypericum*, the observed size divergence is most likely due to differential accumulation of intergenic spacer sequences, a process that enables rapid structural plasticity without impairing essential mitochondrial respiratory functions (Gualberto and Newton, 2017).

### 4.2 Contribution of repetitive sequences and MTPTs to mitogenome expansion

Repetitive sequences in plant mitogenomes are key drives of homologous recombination, contributing to genome evolution and extensive structural rearrangements (Maréchal and Brisson, 2010; Gualberto and Newton, 2017). Repeat-mediated recombination can generate highly dynamic mitochondrial conformations (Cole et al., 2018; Zhong et al., 2022), including circular forms in *Melia azedarach* L. (Hao et al., 2024), linear molecules in *Haematoxylum campechianum* L. (Shen et al., 2025), or multichromosomal structures in *Lilium tsingtauense* Gilg (Qu et al., 2024). In the *H. ascyron* mitogenome, we identified a putative recombinationally active repeat that likely mediates genomic reconfiguration, a phenomenon also reported in *Morus notabilis* C.K.Schneid (Liu et al., 2024a).

Both *H. ascyron* and *H. perforatum* contained three categories of repetitive sequences, SSRs, tandem repeats, and dispersed repeats, widely distributed across their mitogenomes. Among the SSRs, six motif types were detected, with monomeric repeats being the most abundant. This pattern mirrors observation in *Terniopsis yongtaiensis* (Zhang et al., 2024b) and *Hevea* species (Niu et al., 2024). Dispersed repeats were dominated by forward and palindromic types, consistent with those in closely related Malpighiales such as *T. yongtaiensis*, *Calophyllum soulattri* (Cadorna et al., 2024), and *Viola diffusa* (Zhang et al., 2024a), suggesting that repeat proliferation in this order may be shaped by conserved evolutionary constraints.

A total of 22 and 25 MTPTs were identified in *H. ascyron* and *H. perforatum*, respectively, accounting for 24,248 bp (5.9%) and 22,228 bp (4.6%) of their mitogenomes. These proportions are comparable to *Calophyllum soulattri* (4.6%), but lower than in *Terniopsis yongtaiensis* (14.6%), and higher than in *Garcinia mangostana* (1.7%). Such variation reflects lineage-specific dynamics of plastid-to-mitochondrial DNA transfer, a widespread hallmark of angiosperm mitogenomes (Chen et al., 2025). Notably, *H. ascyron* incorporated multiple plastid-derived tRNAs, including *trnC-GCA*, *trnD-GUC*, *trnI-CAU*, *trnM-CAU*, *trnS-GGA*, *trnV-GAC*, and *trnW-CCA*, as well as a partial rrn18 gene. In contrast, *H. perforatum* lacked *trnC-GCA* but uniquely retained *trnH-GUG*. Most MTPTs originated from the plastome large single-copy (LSC) region, consistent with observations in *T. yongtaiensis*. Furthermore, two shared “hotspots” (*trnD-GUC* and *psaB-psaA*) were detected across *H. ascyron*, *H. perforatum*, and *T. yongtaiensis*, indicating that plastid DNA integration may be influenced by sequence-specific biases (Wang et al., 2017; Nhat Nam et al., 2024). The presence of uncharacterized MTPTs further suggests potential functional innovation (Wang et al., 2012). Collectively, these findings highlight the important contribution of MTPTs to mitogenome expansion and structural diversification within *Hypericum*.

### 4.3 Distribution patterns and functional significance of mitochondrial RNA editing

RNA editing is an essential post-transcriptional mechanism in plant mitochondria, restoring conserved codons and ensuring proper mitochondrial protein function (Small et al., 2020; Knoop, 2023). In angiosperms, cytosine-to-uracil (C-to-U) conversions predominate, although uracil-to-cytosine (U-to-C) editing is widespread in other plant lineages, including hornworts, lycophytes, and ferns (Kwok van der Giezen et al., 2025). While the editing mechanism is broadly conserved, the number and distribution of RNA editing sites vary markedly among taxa, with most edits occurring at the first and second codon positions (Covello and Gray, 1993).

In *Hypericum*, 316–324 RNA editing sites were predicted, exhibiting a strong positional bias toward the fist (109–112) and second (187–192) codon positions, with relatively fewer at the third (20–23). This pattern is consistent with other angiosperms and reflects the primary role of RNA editing in correcting non-synonymous mutations (Edera et al., 2018). Among examined PCGs, *ccmC*, *mttB*, *nad2*, and *nad4* harbored the highest number of editing sites, similar to observations in *Terniopsis yongtaiensis* (*ccmB*, *ccmC*, *ccmFn*, *nad2*, *nad4*). The *nad4* gene contained the largest number of editing sites in *Hypericum* (28), comparable to related Malpighiales such as *T. yongtaiensis* (25 sites), *Calophyllum soulattri* (35 sites), and *Garcinia mangostana* (35 sites), suggesting evolutionary conservation in editing intensity among key respiratory genes.

Despite these overall similarities, noticeable interspecific differences were observed. For example, the *ccmC* gene in *H. pulchrum* contained 29 editing sites, compared with 24–25 in other *Hypericum* species, a trend also reported in other plant lineages (i.e., *Quercus* L.; Song et al., 2025). Editing sites in *cox3* were predicted exclusively in *H. pulchrum*, reminiscent of lineage-specific editing loss observed in Amaryllidaceae and Iridaceae, likely attributable to recombination and reverse transcription involving edited transcripts (Lopez et al., 2007).

RNA editing also contributes to the creation of functional start and stop codons (Small et al., 2020; Hao et al., 2021). In *Hypericum*, this included C-to-U conversions such as the edited start codon of *nad1* (ACG→ATG) and the edited stop codon of *ccmFC* (CGA→TGA). U-to-C editing can perform equivalent functions o in other plant lineages, particularly in early vascular plants (Kwok van der Giezen et al., 2025). Similar regulatory roles have been reported in *Azolla* Lam. and *Salvinia* Ség. (Li et al., 2018), underscoring the fundamental importance of RNA editing in mitochondrial gene expression.

### 4.4 Phylogenetic relationships and inter-organellar discordance

Phylogenetic analysis based on shared mitochondrial PCGs from 30 species across Malpighiales, Celastrales and Sapindales recovered a close relationship between Hypericaceae and Podostemaceae, consistent with established taxonomic frameworks (Wurdack and Davis, 2009; Xi et al., 2012; APG IV, 2016). However, discrepancies emerged between mitochondrial and plastid phylogenies within *Hypericum*. In the plastid tree, *H. hirsutum* and *H. pulchrum* formed a well-supported clade, in agreement with previous plastid and nrITS-based studies (Meseguer et al., 2013; Nürk et al., 2013). In contrast, the mitochondrial phylogeny placed *H. pulchrum* closer to *H. perforatum*. Morphological characters support the plastid/nuclear topology: both *H. hirsutum* and *H. pulchrum* belong to sect. *Taeniocarpium* and share traits such as sepals with gland-fringed margins (Robson, 2010), whereas *H. perforatum* has fewer or no intramarginal black glands (Li and Robson, 2007). These observations indicate that mitochondrial topology alone does not fully reflect the species relationships inferred from morphology and plastid/nuclear markers.

Similar inter-organellar discordance has been reported in other angiosperm lineages, including Brassicaceae (Dominicus et al., 2025), *Dalbergia odorifera* T.C.Chen (Hong et al., 2021), *Gossypium* L. (Kong et al., 2025), and *Potentilla* L. (Xue et al., 2024). Such conflicts are often attributed to hybridization, incomplete lineage sorting, horizontal gene transfer, or differences in substitution rates (Lin et al., 2025). Although plant mitochondrial genomes evolve slowly, with synonymous substitution rates approximately one-third and one-sixteenth those of plastid and nuclear genomes, respectively (Drouin et al., 2008; Smith and Keeling, 2015), their conservative nature makes them valuable for resolving deep evolutionary relationships (Qiu et al., 2010). Moreover, mitogenomic features such as RNA editing profiles, repeat content, and GC composition can provide independent phylogenetic signals (Nie et al., 2025). Integrating mitogenomic, plastid, and nuclear data thus enables a more comprehensive understanding of the complex evolutionary history of *Hypericum*.

## 5 Conclusions

This study reports the complete mitochondrial genomes of *Hypericum ascyron* and *H. perforatum* and presents the first comparative mitogenomic analysis within the genus. Although both species retain a conserved set of mitochondrial protein-coding genes, they exhibit notable variation in genome size, largely driven by differences in intergenic spacer sequences and plastid-derived DNA insertions. The identified repetitive sequences and MTPTs provide new insights into the structural dynamism of *Hypericum* mitogenomes. Extensive RNA editing events were also detected, including species-specific patterns that may contribute to post-transcriptional regulation and adaptive evolution. Phylogenetic analyses based on mitochondrial genes corroborated the close relationship between Hypericaceae and Podostemaceae within Malpighiales, while revealing discordance between mitochondrial and plastid phylogenies among *Hypericum* species. Together, these findings provide valuable genomic resources and establish a foundation for further investigations into mitochondrial genome evolution, structural variation, and functional adaptation in *Hypericum* and related lineages.

## Data Availability

The raw sequencing data generated in this study have been deposited in the Genome Sequence Archive at the National Genomics Data Center (NGDC), China National Center for Bioinformation (CNCB), Beijing Institute of Genomics, Chinese Academy of Sciences, under accession number CRA035721 (https://ngdc.cncb.ac.cn/gsa/) (CNCB-NGDC Members and Partners, 2025). Additionally, the data reported in this study have also been deposited in the GenBase (Bu et al., 2024) at NGDC/CNCB under accession number C_AA132761.1–C_AA132762.1 and C_AA133240.1–C_AA133242.1 (https://ngdc.cncb.ac.cn/genbase). Annotations of the mitochondrial and plastid genomes for the other four *Hypericum* species from the Darwin Tree of Life (DToL) project are available in Figshare (https://doi.org/10.6084/m9.figshare.31045846).
